# Supplementary material for: Carissa macrocarpa Leaves Polar Fraction Ameliorates Doxorubicin-Induced Neurotoxicity in Rats via Downregulating the Oxidative Stress and Inflammatory Markers
Source: Pharmaceuticals (Basel). 2021 Dec 14;14(12):1305. doi: 10.3390/ph14121305 (PMC8709457; doi:10.3390/ph14121305)
Supplement: Supplementary file 1 [file pharmaceuticals-14-01305-s001.zip › pharmaceuticals-1481316-supplementary.pdf]

Carissa macrocarpa leaves polar fraction ameliorates doxorubicin-induced neurotoxicity in rats via downregulating the oxidative stress and inflammatory markers

Mohamed A. A. Orabi [1], Heba M. A. Khalil[2,*], Mohamed E. Abouelela[3], Dalia Zaafar[4], Yasmine H. Ahmed[5], Reham A. Naggar[6], Hamad S. Alyami[7], El-Shaymaa Abdel-Sattar[8], Katsuyoshi Matsunami[9] and Dalia I. Hamdan[10,]**

1 Department of Pharmacognosy, College of Pharmacy, Najran University, Najran 55461, Kingdom of Saudi Arabia.

2 Department of Veterinary Hygiene and Management, Faculty of Veterinary Medicine, Cairo University, Giza, 12211, Egypt

3 Department of Pharmacognosy, Faculty of Pharmacy, Al-Azhar University, Assiut-branch, Assiut 71524, Egypt

4 Pharmacology and Toxicology Department, Faculty of Pharmacy, Modern University for Information and Technology

5 Cytology and Histology Department, Faculty of Veterinary Medicine, Cairo University, Giza, 12211, Egypt.

6 Department of pharmacology and Toxicology, college of pharmacy and Drug Manufacturing, Misr University of Science and Technology (MUST), 6th October, Giza 12566, Egypt

7 Department of Pharmaceutics, College of Pharmacy, Najran University, Najran 55461, Kingdom of Saudi Arabia

8 Department of Medical Microbiology and Immunology, Faculty of Pharmacy, South Valley University, 83523, Qena, Egypt

9 Department of Pharmacognosy, Graduate School of Biomedical and Health Sciences, Hiroshima University, 1-2-3 Kasumi, Minami-Ku, Hiroshima 734-8553, Japan

10 Department of Pharmacognosy and Natural Products, Faculty of Pharmacy, Menoufia University, Shibin Elkom, 32511, Egypt

*Corresponding author:
Heba M. A. Khalil,
Department of Veterinary Hygiene and Management, Faculty of Veterinary Medicine, Cairo University, Giza, 12211, Egypt, Mobile: +201013666331. E-mail: heba.ali315@gmail.com.
**Corresponding author:
Dalia. I. Hamdan,
Department of Pharmacognosy and Natural Products, Faculty of Pharmacy, Menoufia University, Shibin Elkom, 32511, Egypt, Mobile: +201289699978. E-mail: masmn12345.1973@gmail.com.

Supplementary Material

List of contents

No	Content	Page
**Figure S1**	Structures of identified compounds and their isomers evaluated by molecular docking simulation	S2
**Figure S2**	Continue structures of identified compounds and their isomers evaluated by molecular docking simulation	S3
**Figure S3**	Continue structures of identified compounds and their isomers evaluated by molecular docking simulation.	S4
**Figure S4**	TIC of *C. macrocarpa* polar fraction of the leaves using UPLC-ESI-MS/MS in negative ionization mode.	S4
**Table S1**	Docking score of detected polyphenolics and their isomers against TACE enzyme	S5
**UPLC-ESI-MS-MS metabolites characterization**		S6-S8
**References**		

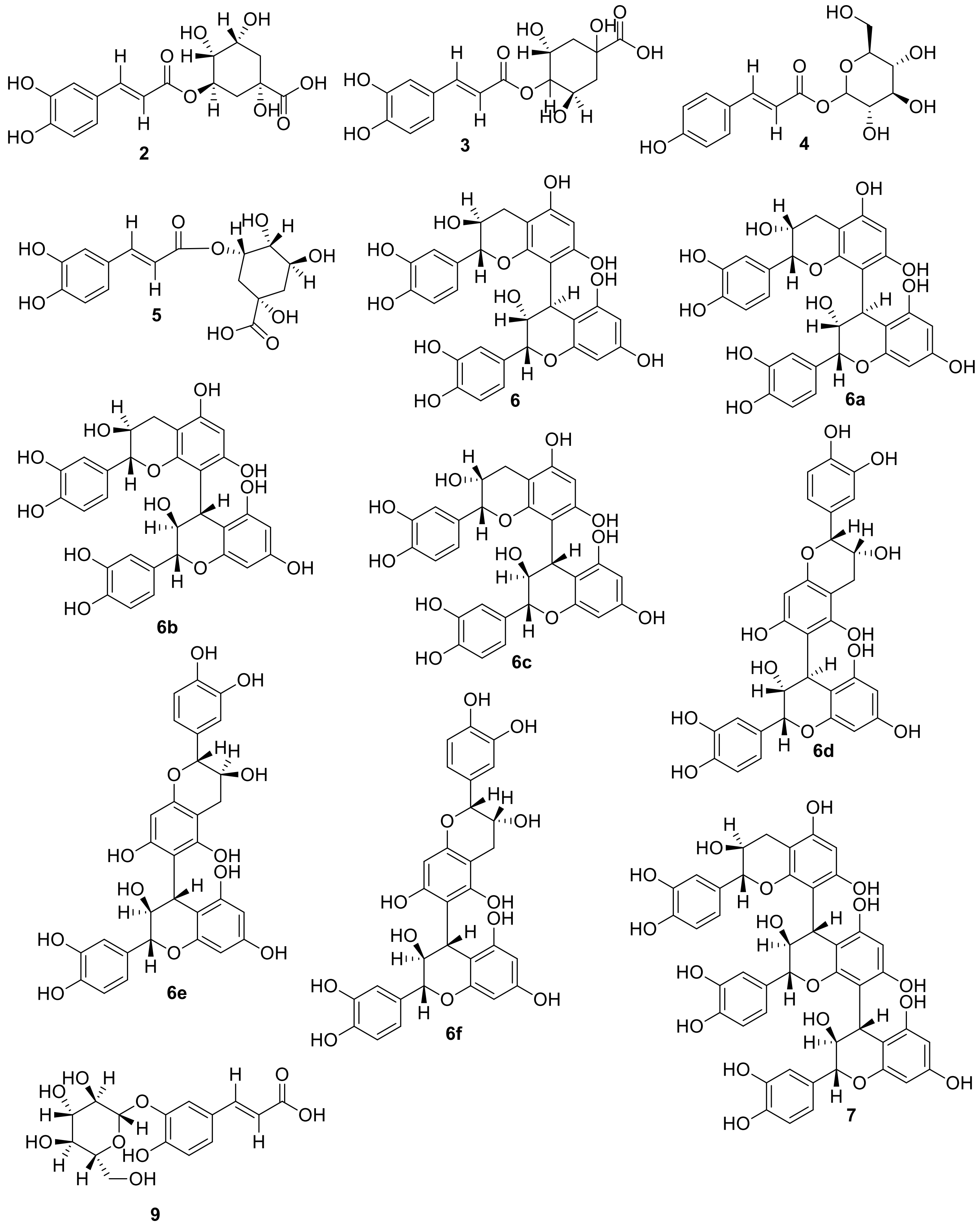

Figure S1. Structures of compounds evaluated by molecular docking simulation.

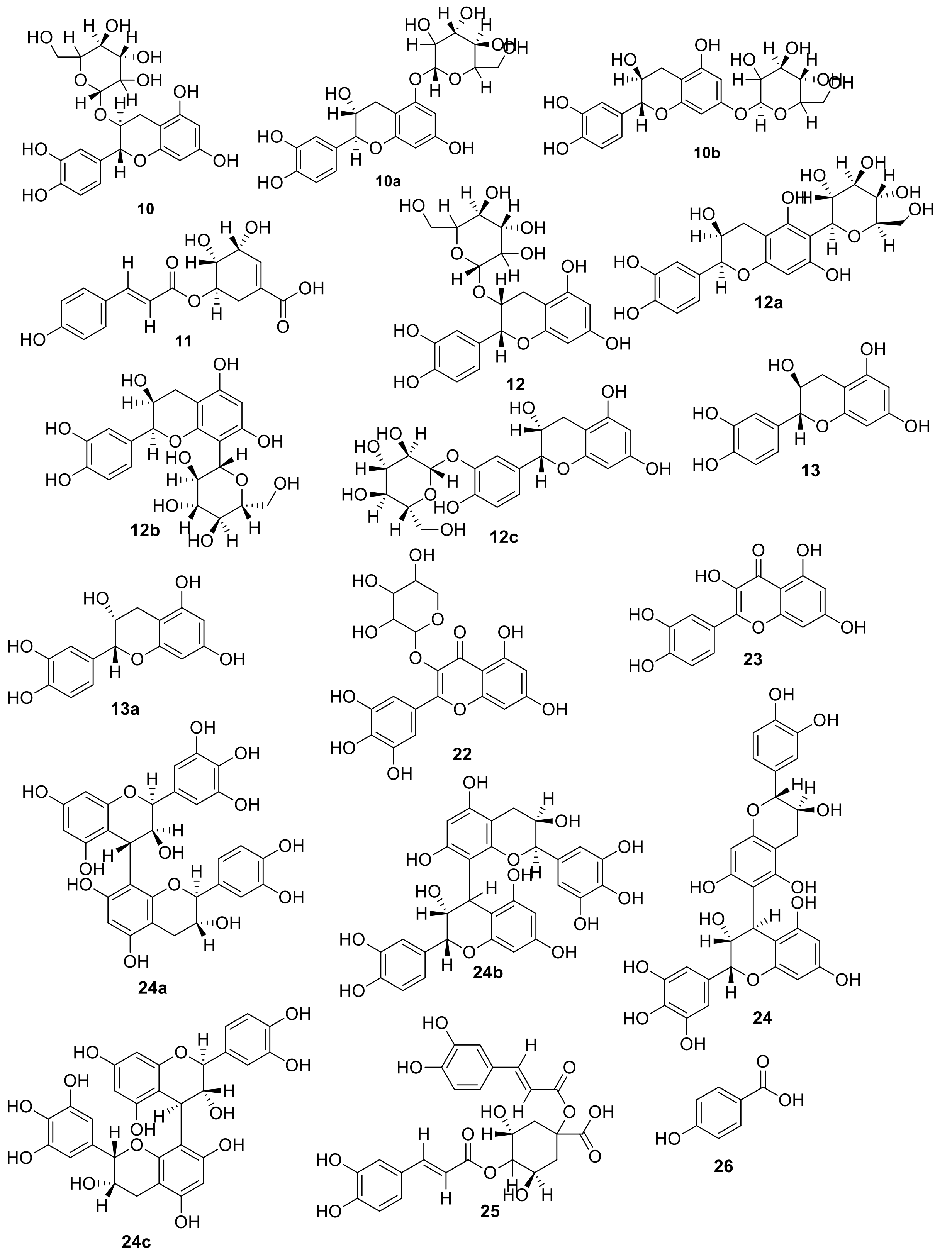

Figure S2. Structures of compounds evaluated by molecular docking simulation: Continued

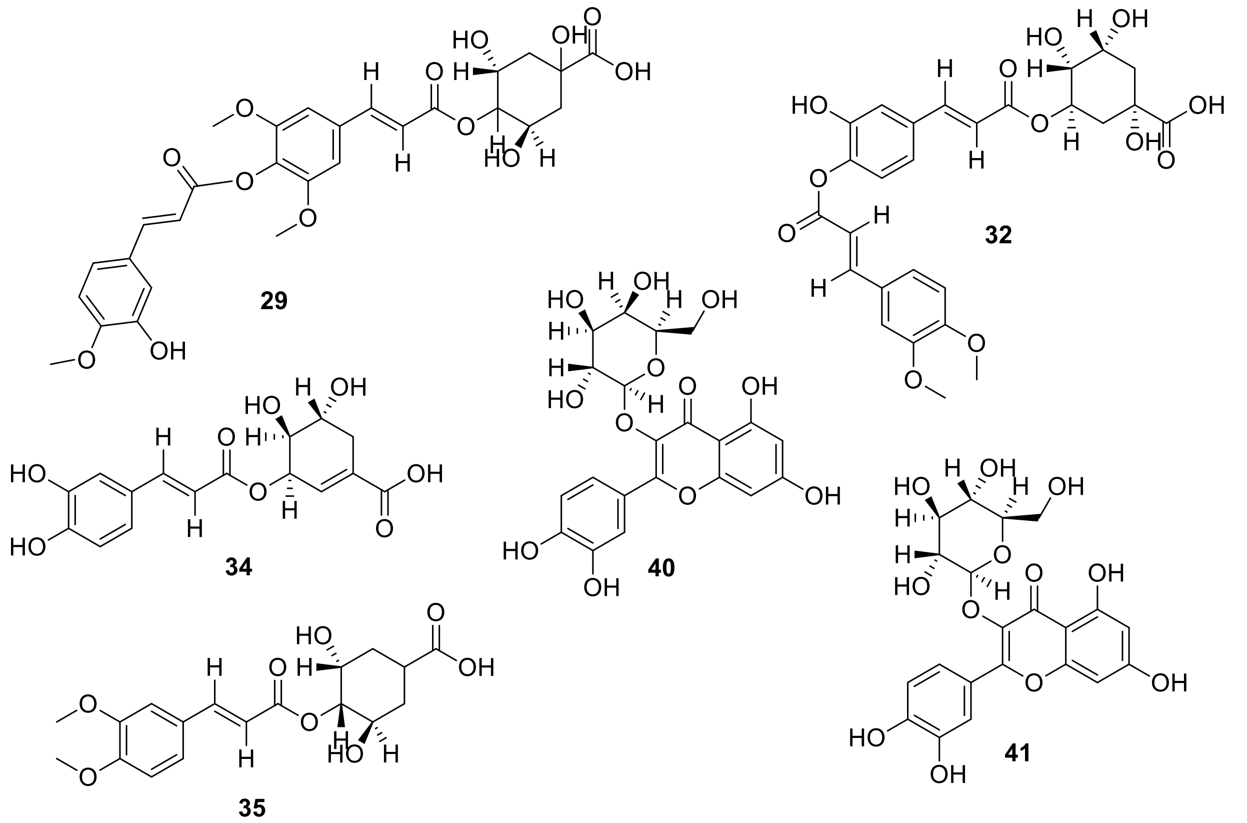

Figure S3. Structures of compounds evaluated by molecular docking simulation: Continued

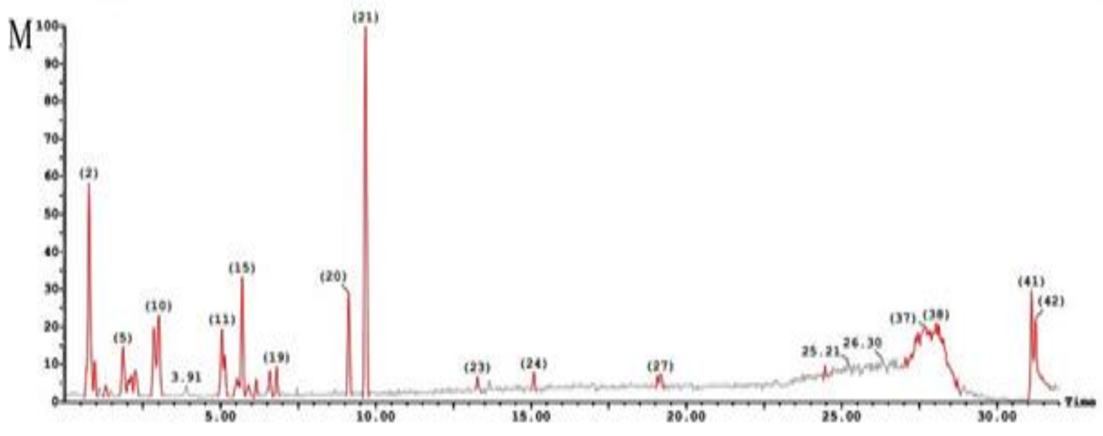

Figure S4. TIC of *C. macrocarpa* polar fraction of the leaves using UPLC-ESI-MS/MS in negative ionization mode.

Table S1. Docking score of detected polyphenolics and their isomers against TACE enzyme

No.	Compound Name	TNF-activating converting enzyme (TACE)	
		Score (kcal/mol)	RMSD refine (Å)
**2**	Chlorogenic acid	-12.6813	0.91
**3**	Cryptochlorogenic acid	-13.3260	0.93
**4**	coumaroyl-5-*β*-glucose	-11.9755	1.07
**5**	5-*O*-Caffeolyquinic acid	-13.6537	0.96
**6**	Procyanidin Bl	-16.2139	2.66
**6a**	Procyanidin B2	-17.5405	1.82
**6b**	Procyanidin B3	-13.9250	1.60
**6c**	Procyanidin B4	-17.5085	0.84
**6d**	Procyanidin B5	-19.9566	1.77
**6e**	Procyanidin B6	-21.9152	1.25
**6f**	Procyanidin B8	-17.0187	1.92
**7**	Procyanidin C2	-13.2742	1.80
**9**	Caffeic acid 3-glucoside	-12.8895	1.44
**10**	Catechin 3-*O*- *β* -D-glucopyranoside	-18.5367	1.69
**10a**	Catechin 5-*O*- *β* -D-glucopyranoside	-17.1693	1.31
**10b**	Catechin 7-*O*- *β* -D-glucopyranoside	-15.3866	1.57
**11**	4-*O*-*p*-Coumaroylshikimic acid	-11.6970	1.24
**12**	Epicatechin 3-*O*- *β* -D-glucopyranoside	-20.6389	1.34
**12a**	Epicatechin 6-C-glucoside	-19.2057	1.30
**12b**	Epicatechin 8-C-glucoside	-19.1597	0.93
**12c**	Epicatechin-3'-*O*-glucoside	-14.7443	1.74
**13**	Catechin	-14.2871	1.02
**13b**	Epicatechin	-14.1969	0.84
**22**	Myricetin-3-*O*-xyloside	-18.2396	1.15
**23**	Quercetin	-13.1148	1.19
**24a**	Epigallocatechin-(4 *β* -8)-catechin	-18.0768	1.27
**24b**	Epicatechin (4 *β*.8) epigallocatechin	-18.3569	1.97
**24c**	Catechin-(4*α*-8)-(-)-epigallocatechin	-18.0083	1.44
**24**	Epigallocatechin-(4 *β* -6)-(+)-catechin	-14.8778	1.82
**25**	1,4-Dicaffeoylquinic acid	-15.0462	1.10
**29**	Feruloyl-*O*-sinapoylquinic acid	-15.8150	1.08
**32**	Dimethoxycinnamoyl-*O*-caffeoylquinic acid	-13.6193	1.00
**34**	3-Caffeoylshikimic acid	-15.6133	0.89
**35**	Dimethoxycinnamoylquinic acid	-13.1423	1.48
**40**	Hyperoside	-19.9102	1.47
**41**	Isoquercetin	-16.0728	1.10

UPLC-ESI-MS-MS metabolites charctrization

Organic phenolic acids and their derivatives

Hydroxybenzoic acid (**26**) and syringic acid (**28**) were detected at different retention times as reported in Table 1. Compounds **2**, **3**, **5**, and **34** that showed a molecular ion at *m/z* 353 were detected at retention times 1.87, 2.03, 2.16, 15.06, and 16.52, respectively. Compounds **2** and **5** produced MS^2 fragments at *m/z* 191 ([quinic acid−H]), 179 ([Caffeic acid−H]), 161 ([caffeic acid−H_2O−H]), hence, assigned as 3-O-caffeolyquinic acid and 5-O-caffeolyquinic acid, respectively. Where 4-O-caffeolyquinic acid was designated for compound **3** with characteristic MS^2 fragments at *m/z* 191, 173 [191−H_2O], and 161 [1].

Additionally, 3-O-caffeoylshikimic acids has been proposed for **34**, depending upon MS^2 fragments (at *m/z* 179, 161) and published data [2]. Another caffeolyquinic acid derivative, diacetoxy-5-methoxyphenyl) acroyl-O-*p*-coumaroyl-O-caffeoylquinic acid (**30**) was identified by the aid of MS, MS^2 data (Table 1), and reported literature [3].

Other hydroxycinnamate derivatives such as 5-O-*p*-coumaroylshikimic and 4-O-*p*-coumaroylshikimic acids have been suggested for peaks **8** and **11**. MS^2 fragmentation exhibited a base peak at *m/z* 163 due to [*p*-coumaric acid−H_2O], as well as other characteristic fragments [3]. Moreover, peak **25**, which was detected at retention time 6.58 showed deprotonated molecular ion at *m/z* 515 and distinctive fragments at *m/z* 353 [M−162] and 179 that are matched with the reported literature of dicaffeoylquinic acid (**25**). Feruloyl-O-sinapoylquinic acid (**29**) was designated for peak detected at *m/z* 573 $[M-H]^-$, based on MS^2 fragments (397 [M−ferulic acid−H], 223 [sinapic acid−H], 173 [quinic acid−H_2O−H]) as compared with the literature [2]. Besides, compounds, dimethoxycinnamoyl-O-caffeoylquinic acid (**32**) and its isomer (**33**) were observed at different retention times 13.30 and 15.66 but exhibited similar deprotonated molecular ions at *m/z* 543 and characteristic fragment ions at *m/z* 353 [M−dimethoxycinnamoyl−H_2O] and 173 that attributed to dimethoxycinnamoyl moiety. In addition, dimethoxycinnamoylquinic acid was suggested for peak **35** that was detected in positive ionization mode at *m/z* 383 (R_t 16.59) [2]. Moreover, hydroxycinnamate glycosides were detected in the current study at retention times 2,13 and 2.87, that are corresponding to peaks **4** and **9**, respectively. Where peaks **4** was suggested to be coumaric acid hexoside, due to molecular ions at *m/z* 325 $[M-H]^-$ which produced fragment ions at *m/z* 187, 163, and 145 in accordance with the literature [4]. Also, caffeic acid hexoside was assigned for peak **9** depending upon its molecular ion at *m/z* 343 $[M+H]^+$ with distinctive fragments at 326, 311, and 285, which in agreement with the reported data [5].

Flavonoids aglycone and glycosides

Flavonoids are considered one of the major polyphenolics detected in the polar fractions of the species under investigation. Peak **14** and **15** provided the same deprotonated molecular ion at *m/z* 755 $[M-H]^-$ with different MS^2 fragments, which were 593 [M−162, sugar unit] and 285 [M−308, rutinoside unit] for peak **14** that assigned as kaempferol-O-hexoside-O-rutinoside. However, quercetin-O-deoxyhexoside-O-deoxyhexosyl-hexoside was proposed for peak **15** that produced fragment ions at m/z 609 [M−146, deoxyhexosyl unit] and 301 $[M-146-308]^-$, deoxyhexosyl and rutinoside units]. Other kaempferol derivatives were kaempferol-O-

deoxyhexoside-O-deoxyhexosyl-hexoside isomers, which were designated for peaks **17** and **18**, respectively. Both compounds provided the same molecular ions (*m/z* 739 $[M{-}H]^{-}$) and MS^2 fragmentation patterns (*m/z* 593 and 285). Moreover, peaks **19** and **20** were tentatively identified as quercetin-O-deoxyhexosyl-hexoside isomers based on their molecular ion at *m/z* 609 $[M{-}H]^{-}$ and MS^2 fragment at *m/z* 301 due to loss of rutinoside unit (−308 amu). Also, the other isomer exhibited fragment ion at *m/z* 465 in positive ionization mode due to loss of deoxyhexosyl unit (−146 amu). All the previous results were confirmed upon comparison with the literature [1].

Additionally, compounds **40** and **41** belong to quercetin derivatives and have been identified as quercetin-3-O-galactoside, and quercetin-3-O-glucoside, respectively, as depicted from their MS, MS^2 data and comparison with literature [6]. Where, compounds **40** and **41** provided the same molecular ion at *m/z* 465 $[M{+}H]^{+}$ and MS^2 fragments at *m/z* 301, 300, 257, 255, 229, 179, and 151.

Furthermore, compound **22** is belonging to flavonoid glycoside [3] that was tentatively identified as myricetin pentoside [7] based on its molecular ions at *m/z* 449 $[M{-}H]^{-}$. Myricetin pentoside showed characteristic MS^2 fragment at 317 [M−132]. Finally, flavonoid aglycone such as quercetin has been proposed for peaks **23**.

Flavan-3-ol derivatives

C. macrocarpa is considered a main source of falvan-3-ol bioactive metabolites. From these interesting molecules, compound **13** has been identified as (epi) catechin based on a molecular ion at *m/z* 289 $[M{-}H]^{-}$, MS^2 fragmentation (*m/z* 245, 205, 203, 187, 179, and 161) and comparison with literature [8]. Furthermore, compounds **6**, **7**, **21,** and **31** belong to (epi)catechin derivatives of B-type; thus, (epi)catechin dimer was assigned for peaks **6**, **21**, and **31** based on deprotonated molecular ion at *m/z* 577 $[M{-}H]^{-}$, MS^2 fragmentation (*m/z* 425, 289) and comparison with the literature [1]. However, peak **7** that showed pseudo-molecular ion at *m/z* 865 $[M{-}H]^{-}$, and fragmentation patterns at *m/z* 451, 425, 407, and 289, were tentatively identified as (epi)catechin trimer [1].

Moreover, compounds **10**, **12**, and **16** provided the same deprotonated molecular ion at *m/z* 451 $[M{-}H]^{-}$ and different MS^2 fragments at *m/z* 408, 393, 351, 337, 301, 273, and 245 for compound **10** that suggested to be catechin 3-O-glucoside. However, epicatechin 3-O-glucoside and its isomer were designated for compounds **12** and **16** with MS^2 fragments at *m/z* 391, 343, 301, 287, 273, and 247 [9].

Compounds **24** and **27** were detected at the same molecular ion at *m/z* 593 $[M{-}H]^{-}$ and showed MS^2 fragments at *m/z* 557, 467, 441, 425, 407, and 289, hence proposed to be (epi)gallocatechin-(epi)catechin [7].

Sterols and triterpenes

Stigmasterol (**39**) was detected methanol soluble fraction [10,11]. Furthermore, the current study detected several triterpenoid aglycones at different retention times, such as compounds **36**, **37**, and **38**. Where compounds **36**, **37**, and **38** provided the same molecular ion at *m/z* 455 $[M{-}H]^{-}$,

MS^2 fragment ions (*m/z* 439, 419, 411, 410, 407, and 397) and designated as ursolic acid, carissic acid, and oleanolic acid upon comparison with literatures [12–15].

Miscellaneous

Lignans such as carinol (*m/z* 377 $[M-H]^-$ and MS^2 at 333, 271, 257, 163, and 119) was assigned for compound (**1**) depending on a comparison of its data with that previously reported [13]. Compound **42** with *m/z* 621 $[M-H]^-$ and distinctive fragment ion at 501 was assigned as 2(R)-26-([(2E)-3-(4-hydroxy-3-methoxyphenyl)-1-oxo-2-propen-1-yl]oxy)-2,3-dihydroxypropylester [16]. Finally, compound **43** that detected at retention time at 31.25 possessed molecular ion at *m/z* 429 $[M-H]^-$ and 431 $[M+H]^+$ with characteristic MS^2 fragments at m/z 205, 191, 177, 149, 121, hence assigned as α-tocopherol [17].

References

1. Souilem F, Dias MI, Barros L, Calhelha RC, Alves MJ, Harzallah-Skhiri F, et al. Amantagula fruit (Carissa macrocarpa (Eckl.) A. DC.): nutritional and phytochemical characterization. Plant Foods Hum Nutr. 2019;74(1):76–82.

2. Jaiswal R, Matei MF, Subedi P, Kuhnert N. Does roasted coffee contain chlorogenic acid lactones or/and cinnamoylshikimate esters? Food Res Int. 2014;61:214–27.

3. Ben Said R, Hamed AI, Mahalel UA, Al-Ayed AS, Kowalczyk M, Moldoch J, et al. Tentative characterization of polyphenolic compounds in the male flowers of Phoenix dactylifera by liquid chromatography coupled with mass spectrometry and DFT. Int J Mol Sci. 2017;18(3):512.

4. Alakolanga A, Siriwardane A, Kumar NS, Jayasinghe L, Jaiswal R, Kuhnert N. LC-MSn identification and characterization of the phenolic compounds from the fruits of Flacourtia indica (Burm. F.) Merr. and Flacourtia inermis Roxb. Food Res Int. 2014;62:388–96.

5. Pereira E, Pimenta AI, Calhelha RC, Antonio AL, Verde SC, Barros L, et al. Effects of gamma irradiation on cytotoxicity and phenolic compounds of Thymus vulgaris L. and Mentha x piperita L. LWT-Food Sci Technol. 2016;71:370–7.

6. Lin L-Z, Chen P, Harnly JM. New phenolic components and chromatographic profiles of green and fermented teas. J Agric Food Chem. 2008;56(17):8130–40.

7. Jaiswal R, Jayasinghe L, Kuhnert N. Identification and characterization of proanthocyanidins of 16 members of the Rhododendron genus (Ericaceae) by tandem LC–MS. J Mass Spectrom. 2012;47(4):502–15.

8. Kang J, Price WE, Ashton J, Tapsell LC, Johnson S. Identification and characterization of phenolic compounds in hydromethanolic extracts of sorghum wholegrains by LC-ESI-MSn. Food Chem. 2016;211:215–26.

9. Yuzuak S, Ballington J, Xie D-Y. HPLC-qTOF-MS/MS-based profiling of flavan-3-ols and dimeric proanthocyanidins in berries of two muscadine grape hybrids FLH 13-11 and FLH 17-66. Metabolites. 2018;8(4):57.

10. Petta V, Gkiozos I, Strimpakos A, Syrigos K. Histones and lung cancer: are the histone deacetylases a promising therapeutic target? Cancer Chemother Pharmacol. 2013;72(5):935–52.

11. Zhou Y-X, Xin H-L, Rahman K, Wang S-J, Peng C, Zhang H. Portulaca oleracea L.: a review of phytochemistry and pharmacological effects. BioMed Res Int. 2015;2015.

12. Moodley R, Chenia H, Jonnalagadda SB, Koorbanally N. Antibacterial and anti-adhesion activity of the pentacyclic triterpenoids isolated from the leaves and edible fruits of Carissa macrocarpa. J Med Plants Res. 2011;5(19):4851–8.

13. Patel S. Food, pharmaceutical and industrial potential of Carissa genus: an overview. Rev Environ Sci Biotechnol. 2013;12(3):201–8.

14. Kaunda JS, Zhang Y-J. The genus Carissa: An ethnopharmacological, phytochemical and pharmacological review. Nat Prod Bioprospecting. 2017;7(2):181–99.

15. Jiao Q-S, Xu L-L, Zhang J-Y, Wang Z-J, Jiang Y-Y, Liu B. Rapid characterization and identification of non-diterpenoid constituents in Tinospora sinensis by HPLC-LTQ-orbitrap MSn. Molecules. 2018;23(2):274.

16. Yu J-Q, Xian Y-X, Geng Y-L, Wang D-J, Zhou H-L, Wang X. Anti-liver cancer constituents from the thorns of Gleditsia sinensis. Phytochem Lett. 2015;13:343–7.

17. Izhar S, Ahmed D. Carissa opaca: A plant with great potential for future drugs for degenerative and infectious diseases. ChemistrySelect. 2016;1(12):3005–11.
